# Socioemotional development in infants of pregnant women during the COVID-19 pandemic: the role of prenatal and postnatal maternal distress

**DOI:** 10.1186/s13034-022-00458-x

**Published:** 2022-03-31

**Authors:** Gabrielle Duguay, Julia Garon-Bissonnette, Roxanne Lemieux, Karine Dubois-Comtois, Kristel Mayrand, Nicolas Berthelot

**Affiliations:** 1 Departement of Psychology, Université du Québec À Trois-Rivières, 3351 Boulevard des Forges, C.P. 500, Trois-Rivières, Québec, G9A 5H7 Canada; 2 Department of Nursing Sciences, Université du Québec À Trois-Rivières, 3351 Boulevard des Forges, C.P. 500, Trois-Rivières, Québec, G9A 5H7 Canada; 3 Centre d’études Interdisciplinaires Sur le Développement de L’enfant et la Famille, Québec, Canada; 4 Groupe de Recherche et d’intervention Auprès des Enfants Vulnérables et Négligés, Québec, Canada; 5 CERVO Brain Research Center, Québec, Canada; 6 Interdisciplinary Research Center on Intimate Relationship Problems and Sexual Abuse, Québec, Canada; 7 Centre Intégré Universitaire de Santé et de Services, sociaux du Nord-de-l’Île-de-Montréal, Québec, Canada; 8 Departement of Social Sciences, Université Sainte-Anne, 1695, Route 1, Church Point, Nova Scotia, B0W 1M0 Canada

**Keywords:** COVID-19, Perinatal distress, Infant socioemotional development

## Abstract

**Background:**

An upsurge in psychological distress was documented in pregnant women during the COVID-19 pandemic. We investigated with a longitudinal design whether prenatal and postnatal maternal distress during the COVID-19 pandemic was associated with lower infant socioemotional development.

**Methods:**

Pregnant women (N = 468, M_*age*_ = 30,00, 97.6% White) were recruited during the first COVID-19 mandatory lockdown in Quebec, Canada, from April 2nd to April 13th 2020 and were re-contacted at two months postpartum to complete self-reported measures of general (i.e. not specifically related to the COVID-19 pandemic) anxio-depressive symptoms and infant development. Structural equation modeling analyses were performed using maximum likelihood parameter estimation.

**Results:**

Higher maternal prenatal distress significantly contributed to poorer infant socioemotional development. A mediation model showed that postnatal distress significantly mediated the association between prenatal distress and infant socioemotional development, whereas the direct effect of prenatal distress was no longer significant. Prenatal and postnatal maternal distress accounted for 13.7% of the variance in infant socioemotional development.

**Conclusion:**

Our results call for special means of clinical surveillance in mothers and for innovative (online) interventions aiming to support maternal mental health during pregnancy and after delivery.

**Supplementary Information:**

The online version contains supplementary material available at 10.1186/s13034-022-00458-x.

## Background

The threat caused by the Coronavirus disease 2019 (COVID-19) to physical health in adult populations, the uncertainty and upheaval engendered by the pandemic, and the public health measures of social distancing and lockdown have greatly challenged the psychological health in all populations [1, 2]. Pregnant women may represent a particularly high-risk group for psychological distress during the COVID-19 pandemic given that the perinatal period is a time of heightened vulnerability for mental health [3]. Moreover, the particular threat of a COVID-19 infection during pregnancy [4] and a restrained social support during the pandemic [5] may have exacerbated the stress experienced by mothers and thus weakened their mental health [6–8]. As a result, a significant increase in psychological distress was documented among pregnant women during the pandemic [9–13], especially in the form of depressive and anxiety symptoms [5, 14–17].

This upsurge in prenatal distress in pregnant women during the COVID-19 pandemic is alarming considering that anxiety and depressive symptoms during pregnancy have been associated with poorer outcomes in offspring, such as early gestational age, lower birth weight, and developmental delays [18–21]. A meta-analysis performed before the COVID-19 pandemic reported that the odds of having a child with behavioral difficulties were 1.63 times greater for pregnant women reporting symptoms of depression or anxiety than for women who did not [22]. For this reason, it has been argued from the beginning of the pandemic that the effect of the COVD-19 pandemic on pregnant women and their newborn should be closely monitored [23].

To our knowledge, few studies have yet documented the association between prenatal maternal distress during the COVID-19 pandemic and infant development [24, 25]. A study by Provenzi et al. [26] showed that greater COVID-19 related maternal stress during pregnancy was significantly associated with higher levels of SLC6A4 gene methylation in offspring at 3-months, which was in turn associated with a lower activity level and a lower expression of pleasure in infants. In another study using the same sample, the authors observed that regulatory capacity of infants was not correlated with maternal prenatal distress but was negatively associated with maternal postnatal distress via higher parenting stress and lower postnatal bonding [27]. These results are in line with previous pre-pandemic studies showing that prenatal and postnatal distress are distinct and complementary contributors to early child development [28]. In the current study, we focused on infant socioemotional development since it was previously shown to be impacted by maternal prenatal distress [22] and since it has been associated with multiple lifetime outcomes such as with executive functioning and academic success during childhood [29, 30], or later with employment, substance use, or mental health in adulthood [31].

The present study aimed to provide preliminary data regarding the contribution of general (i.e. not specifically related to the COVID-19 pandemic) pre- and postnatal maternal distress to early infant development during the COVID-19 pandemic. We first evaluated whether the severity of prenatal distress in mothers that were pregnant during the COVID-19 pandemic was prospectively associated with infant socioemotional development. Since previous studies had shown that prenatal distress in mothers increased the risk of postnatal distress [32, 33], and that postnatal distress would be determinant for infant socioemotional development [34, 35], we next examined whether the association between maternal prenatal distress and infant socioemotional development was mediated by maternal postnatal distress. As a secondary objective, we wanted to confirm the validity of our developmental/longitudinal model and rule out the possibility of postnatal distress being associated with infant development because depressed and anxious mothers were more likely to display a negative appreciation of their infant’s development [36–38]. We thus evaluated whether only offspring of mothers with persisting distress over pregnancy and the postnatal period were different from offspring of mothers without any distress.

## Methods

### Participants and procedure

A sample of 1278 pregnant women was recruited online from social media platforms during the first COVID-19 mandatory lockdown in the Province of Quebec, Canada, from April 2nd to April 13th 2020. Data collection at first assessment took place during the beginning of the pandemic when public health measures became vigorous after March 13th, 2020 (closure of all non-essential businesses; self-isolation; restriction of non-essential activities) and the level of insecurity and fear was high. Inclusion criteria for the first assessment (T1) were: being 18 years or older, having sufficient reading skills to complete self-report instruments, and being pregnant. In a previous publication, we had shown that these participants had higher levels of anxio-depressive, dissociative and post-traumatic symptoms than women that were pregnant prior to the COVID-19 pandemic, as well as higher negative affectivity and lower positive affectivity [9]. All participants with an infant between 6 and 13-weeks (which is the age range targeted by the two-month version of our measure of socioemotional development) when the second assessment (T2) took place (July 13th 2020 to February 19th 2021) and who accepted to be re-contacted (n = 977) were invited to participate by email in the longitudinal follow-up. Exclusion criteria were: having a diagnosis of schizophrenia or bipolar disorder, having experienced major complications during pregnancy, suspicion of neurodevelopmental or genetic problems (ex., Down syndrome, handicap), and extreme preterm birth (28 weeks and less). Out of the 977 participants who were contacted, 429 did not complete the measures (tabulated here as refusals). Of the 548 participants who agreed to participate, 58 completed less than half of the questionnaires, 18 participants did not respond to the outcome measure (infant socioemotional development) and 4 participants completed the measures when their infants were aged < 6 weeks or > 13 weeks and were thus excluded from the analyses. The final sample included 468 women and their infants. Participants’ characteristics are presented in Table 1. Women who did not participate in the follow-up had not been significantly different at the first assessment from participating women in terms of distress, *t*(1716) = 1.779, *p* = 0.075, or depressive symptoms, *t*(1754) = 1.705, *p* = 0.088.

**Table 1:** Characteristics of the sample

	N	%
Trimester of pregnancy at T1 (n = 467)		
1st trimester	69	14.8
2nd trimester	182	39.0
3rd trimester	216	46.2
Maternal education (n = 467)		
High school diploma or less	17	3.6
Collegial or professional training	142	30.4
University degree	308	65.9
Maternal ethnicity (n = 466)		
White	457	98.1
Black	2	0.4
Hispanic	3	0.6
Other	4	0.8
Couple with other parent at T1 (yes)	458	98.5
Family income (n = 464)		
< $35.000 CAD	19	4.1
> $35.000–< $65.000 CAD	52	11.2
> $65.000-–< $95.000 CAD	147	31.7
≥ $95.000 CAD	246	53.0
Financial situation affected by COVID-19 (n = 467)		
No change	226	48.4
Small reduction	177	37.9
Large reduction	64	13.7
Parental status (primiparous) (n = 465)	297	63.9
Infant sex (females) (n = 457)	221	48.4

	M	SD
Maternal age	30.00	3.77
Weeks of pregnancy at T1	25.53	9.11
Gestational age (weeks)	38.97	2.49
Infant age at T2 (weeks)	10.50	2.60
EPDS total score (pregnancy)	8.83	4.79
K10 total score (pregnancy)	21.28	5.79
EPDS total score (2-months)	6.83	4.75
K10 total score (2-months)	19.19	5.62
ASQ-SE:2 total score	22.98	15.43

Measures were completed online at both time points on a secure portal. Informed consent was obtained from all participants. This study was approved by the Institutional Review Board of Université du Québec à Trois-Rivières (#CER-20-266-10.10). The study was conducted in accordance with the ethical standards of the American Psychological Association.

### Measures

Sociodemographic questionnaires were administered during pregnancy (assessing age, gender, marital status, family income) and at 2 months postpartum (assessing changes in the familial situation, delivery complications, and infant age, sex and health status).

Maternal distress at T1 (prenatal) and maternal distress at T2 (postnatal) were each operationalized through a latent variable estimated from two validated and widely used instruments of anxiety and depressive symptoms: the French versions of the Kessler Psychological Distress Scale (K10) [39] and of the Edinburgh Postnatal Depression Scale (EPDS) [40]. The K10 is a 10-item self-report measure using a 5-point Likert scale, ranging from 1 (None of the time) to 5 (All of the time). A higher total score is indicative of greater severity of distress and 76.3% of respondents with scores ≥ 30 would meet the criteria of the 4th edition of the Diagnostic and Statistical Manual of Mental Disorders (DSM-IV) for anxiety, affective, or substance use disorder during a diagnostic interview [41]. Both the English [39] and French [42] versions showed satisfactory psychometric properties and the instrument has been shown to adequately screen for mood and anxiety disorders in pregnant women [43] and postnatally [44, 45]. Cronbach's α for the K10 in this study was 0.86 at T1 and 0.87 at T2. The EPDS is a 10-item self-report measure using a variable 4-point Likert scale ranging between 0 and 3. Higher total scores reflect greater severity of depressive symptoms and 88% of respondents with scores ≥11 would meet the criteria according to some semi-structured interview reference standards [46]. The French [47] and English [40] versions of the EPDS have shown good reliability and validity when used for measuring prenatal and postnatal depressive symptoms [48, 49]. The Cronbach’s alpha for the EPDS in this study was of 0.84 at T1 and 0.85 at T2.

Infant socioemotional development was assessed between 6 and 13 weeks postpartum using the 2-month version of the Ages and Stages Questionnaire: Social-Emotional, second edition (ASQ:SE-2; Squires et al., 2015). It is a 15-item mother-reported measure using a 3-point Likert scale (0 = Rarely or never, 5 = Sometimes, 10 = Most of the time) and an item that allows parents to indicate if the behavior is of concern to them. A higher score indicates poorer socioemotional development. A score between 25 and under 35 suggests that some behaviors are of concern and should be monitored; a score ≥ 35 suggests that further assessments are needed and professional help and services should be sought out. The instrument assesses five domains of socioemotional development: self-regulation, adaptive functioning, affect, social communication and interaction with caregivers [50] and has good internal consistency, sensitivity and specificity [51]. The Cronbach’s alpha for the ASQ:SE-2 in this study was of 0.62, an alpha that is comparable to what was reported in other previous studies using a maternal report of child development [28, 52].

### Data analysis

Pearson correlations were first conducted among variables in order to identify potential confounding variables. Structural equation modeling (SEM) analyses were next performed with MPlus [53] using maximum likelihood parameter estimation to examine the association between maternal distress and infant socioemotional development. A first regression-based model was performed to measure the effect of prenatal distress on infant socioemotional development. We next included maternal postnatal distress as a mediator in the model [54]. The indirect pathway from prenatal distress to infant socioemotional development through postnatal distress was assessed using Bootstrapping with 10,000 bootstrap samples. The adequacy of the model fit was assessed using four indices: a non-significant χ^2^, a root mean square error of approximation (RMSEA) ≤ 0.06, a comparative fit index (CFI) ≥ 0.95, and a standardized root square residual (SRMR) ≤ 0.08 [55, 56]. A minimum of 10 participants per parameter assessed is required for the calculation of a simple structural equation model to achieve the desired statistical power. In this study, 25 parameters were estimated, which means that a minimum of 250 participants was needed [57]. Finally, to rule out the possibility of postnatal distress being associated with infant development only because mothers who are depressed and anxious are more likely to display a negative appreciation of their infant development, we created four groups of mothers according to clinical cut-offs at the K10 and the EPDS: mothers without distress at both time points, mothers with prenatal distress only, mothers with postnatal distress only and women with persisting distress. An ANCOVA was performed, with infant age in weeks as covariate to evaluate whether only offspring of mothers with persisting distress over pregnancy and the postnatal period were different from offspring of mothers without any distress, or whether such differences in the offspring socioemotional development were similarly observed in offspring of mothers with clinical levels of distress only during the postnatal period.

## Results

**Fig. 1:**
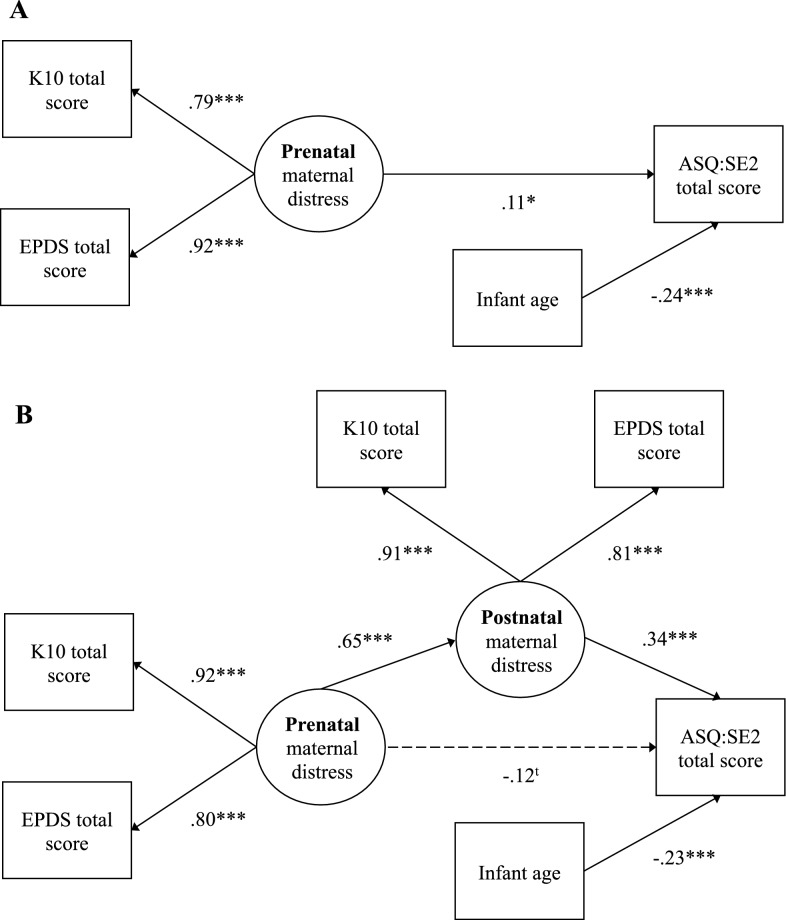
Mediating effect of prenatal maternal distress on infant socioemotional development via postnatal maternal distress. **A** represents the direct effect of maternal prenatal distress on infant socioemotional development, controlling for infant age in weeks. **B** represents the indirect effect of maternal prenatal distress on infant socioemotional development via maternal postnatal distress, controlling for infant age in weeks. *p < .05, ***p < .001, ^t^ *p* = .069

As shown in Table 2, no sociodemographic variables were associated with the outcome, except for the age of the infant in weeks. Thus, it was included as a covariate in all analyses. In the first model (Fig. 1A), the effect of prenatal distress on infant socioemotional development was significant (β = 0.109, *p* = 0.026) and explained 6.5% of the variance in infant socioemotional development. Indices revealed an excellent fit for the model, χ^2^(2, *N* = 468) = 2.621, *p* = 0.270, CFI = 0.998, RMSEA = 0.026 with 90% CI [0.000, 0.099]. In the second model, including maternal postnatal distress as mediator, the indirect effect of prenatal distress on infant socioemotional development via postnatal distress was significant (*b* = 0.646, 95% CI [0.374, 0.993], β = 0.222) and prenatal distress no longer significantly contributed to infant development (β = − 0.118, *p* = 0.069). Indices revealed an excellent model fit, χ^2^(7, *N* = 468) = 11.720, *p* = 0.110, CFI = 0.995, SRMR = 0.028, RMSEA = 0.038 with 90% CI [0.000, 0.075]. Prenatal and postnatal maternal distress accounted for 13.7% of the variance in infant socioemotional development.

**Table 2:** Correlations between study variables

Variables^a^	1	2	3	4	5	6	7	8	9	10	11	12	13	14
1. EPDS total score (pregnancy)	–													
2. K10 total score (pregnancy)	0.733**	–												
3. EPDS total score (2 months)	0.434**	0.457**	–											
4. K10 total score (2 months)	0.466**	0.555**	0.736**	–										
5. Total score ASQ:SE-2 ^b^	0.083	0.079	0.233**	0.214**	–									
6. Maternal age	− 0.107*	− 0.126**	− 0.075	− 0.131**	0.022	–								
7. Weeks of pregnancy at T1	0.015	− 0.012	− 0.044	− 0.051	− 0.002	0.043	–							
8. Maternal education	− 0.162**	− 0.141**	− 0.069	− 0.046	− 0.037	0.233**	− 0.062	–						
9. Couple w/ other parent (yes/no)	0.014	0.080	− 0.030	0.054	− 0.034	− 0.073	0.036	0.047	–					
10. Family income	− 0.236**	− 0.208**	− 0.132**	− 0.121**	− 0.085	0.235**	0.025	0.390**	0.144**	–				
11. Financial situation affected by COVID-19	0.153**	0.137**	0.105*	0.062	− 0.027	− 0.082	− 0.009	− 0.128**	0.006	− 0.158**	–			
12. Parental status (primiparous/multiparous)	0.035	− 0.003	0.032	0.001	− 0.061	0.280**	− 0.031	− 0.062	− 0.017	0.058	− 0.032	–		
13. Gestational age	− 0.018	0.020	0.027	0.017	0.034	− 0.029	0.086	0.043	− 0.006	− 0.004	0.065	− 0.028	–	
14. Infant age at T2	0.075	0.048	0.056	0.078	− 0.224**	− 0.01	− 0.222**	0.008	0.079	0.004	0.042	0.127**	− 0.324**	–
15. Infant sex	0.062	0.035	0.106*	0.064	0.014	− 0.142**	− 0.005	− 0.058	− 0.084	− 0.037	− 0.017	− 0.002	− 0.031	− 0.051

*p < 0.05, **p < 0.01

^a^Pearson’s correlations were used to assess associations between continuous variables; Point-Biserial correlations were used to assess associations between dichotomous and continuous variables; Spearman’s correlations were used to assess associations between ordinal and continuous variables.

^b^Correlations between scores of maternal distress and the subscales of the ASQ-SE:2 are provided in the Additional file 1: Table S1.

Similar results were obtained using a categorical score of infant socioemotional development (≥ 35): maternal prenatal symptoms were indirectly and significantly associated with developmental delays via maternal postnatal symptoms, b = 0.084, 95% CI [0.038, 0.131], and only postnatal symptoms had a direct and significant effect on developmental delays, OR = 1.14, 95% CI [1.064, 1.225].

The ANCOVA (see Additional file 2: Table S2) revealed significant differences regarding infant socioemotional development across the four subgroups established using validated cut-offs of depression and distress when controlling for infant age in weeks, *F*(3, 452) = 9.492, *p* < 0.001. Post hoc analyses (Bonferroni correction) showed that only mothers who reached a clinical level of distress at both time points reported poorer infant development (*n* = 62, *M* = 28.205, *SE* = 1.894) than mothers without any distress (*n* = 257, *M* = 21.116, *SE* = 0.929), mean difference = − 7.089, *p* = 0.005, *SE* = − 0.475.

## Discussion

Our findings reveal that maternal prenatal and postnatal distress during the COVID-19 pandemic was associated with poorer infant socioemotional development. Interestingly, the upsurge of psychological distress observed during the COVID-19 pandemic in pregnant women did not exert a direct negative effect on infant socioemotional development beyond and above the effect of maternal postnatal distress. However, women who reported higher prenatal distress were at higher risk of reporting postnatal distress, which in turn predicted poorer socioemotional development in their infant. The association between postnatal maternal distress and developmental problems in infants was also observed using the clinical cut-off of the measure of socioemotional development, which supports the clinical relevance of our findings.

Our results are in line with a recent study showing that COVID-19-related maternal stress during pregnancy was associated with higher postnatal anxiety, which was associated in turn with lower infants regulatory capacity at 3 months [27]. Our results are also in line with a study using a pre-pandemic sample with similar protective factors (high level of education; in a relationship with the other parent) showing that maternal trait anxiety and depression measured three months after childbirth were the most significant predictors of infant negative affect, suppressing the effect of prenatal trait anxiety [58]. Our finding of lower socioemotional development in offspring of mothers with persistent distress may bear significance for later development, considering that a study that followed children from pregnancy up to 10-years old showed that offspring of mothers reporting consistently high levels of anxiety, depression, and perceived stress during the antenatal period, had higher hazard of mental and behavioral disorders than offspring of mothers with moderate or low levels of symptoms [59]. Previous findings have also shown that early socioemotional development is predictive of later adaptation [29, 30, 60].

The associations we observed between maternal pre- and postnatal distress and infant socioemotional development may involve neurodevelopmental, epigenetic and environmental mechanisms. First, during the prenatal period, impaired placental function has been observed in pregnant women with high levels of subjective stress, resulting in a lower expression and activity of 11β-HSD2, an enzyme that plays a critical role in preventing fetal exposure to maternal cortisol [61]. This impaired placental function has been repeatedly associated with poor birth and developmental outcomes in offspring [18, 22, 62–64]. Second, offspring of mothers who expressed greater COVID-19 related stress during pregnancy had higher levels of SLC6A4 gene methylation at 3-months, which was in turn associated with a lower activity level and expression of pleasure, which suggests an epigenetic pathway between maternal prenatal stress during the COVID-19 pandemic and infant development [26]. Third, maternal distress during the perinatal period would increase the risk that the fetus and eventually the infant (via breastfeeding) become exposed to inflammatory markers, which may have deleterious impacts on brain and behavioral development [65, 66].

However, our observation that the effect of prenatal distress on infant development is mediated by the severity of maternal postnatal distress suggests that postnatal adversity is not only more frequent in offspring of mothers who experienced prenatal stress, but may also be determinant in triggering the vulnerability generated by exposure to stress in utero. In this regard, mothers experiencing postnatal depressive and anxiety symptoms may be less emotionally available to respond to their child's needs in a sensitive and contingent manner [67], the kind of behaviors that could have contributed to mitigate the deleterious impacts of maternal prenatal distress. During the COVID-19 pandemic, the difficulties encountered by distressed mothers in caring for their infant may have more definite consequences than usual, considering that public health measures limited the provision of support by significant others. Mothers were then left isolated, deprived of the help required to get better, and all care for the infant was assumed by the parents, even when the parents’ availability was hindered by psychological distress.

One cannot rule out the possibility that women with postpartum distress had cognitive biases that lead to negative perceptions of themselves and their infants [36–38], which may have resulted in inflated scores on the measure of infant socioemotional development. However, our observation that only mothers with persisting distress over pregnancy and the postnatal period reported significantly poorer infant development, and not mothers who reached clinical levels of distress solely at the postnatal assessment, offers some support to our developmental model.

On the one hand, our findings regarding the contribution of higher maternal distress during the COVID-19 pandemic to poorer infant socioemotional development is alarming for two reasons. First, maternal distress has been shown to be frequent during the COVID-19 pandemic [9–11, 16, 68, 69], meaning that a higher number of children than usual will grow up with a mother whose health, availability, interactions, and skills are altered by psychological distress. Indeed, a Canadian national cross-sectional study reported worse mental health in parents of children under the age of 18 and more frequent negative interactions with their children due to the pandemic [70]. Second, the public health measures put in place to limit the transmission of the virus (such as physical distancing, use of masks, limitations of interactions) may deprive infants with early socioemotional delays from opportunities to learn about self-regulation, social-communication, and emotions, which could have contributed to redress their trajectory, an opinion shared by several experts in the field [65, 71].

On the other hand, our findings are also reassuring and hopeful. Indeed, we reported that transitory distress during pregnancy seemed to have little negative effects on infant socioemotional development. This suggests that pregnant women should not overly worry about the repercussions on their child of feeling anxious or depressed, which is somewhat expected when facing challenging life circumstances such as the COVID-19 pandemic, but should consider these symptoms as a warning signal that some help or adaptations may be required. By doing so, they may act to make sure their distress does not become pervasive nor impact the development of their child.

This study presents strengths and limitations. One strength is the use of well-validated instruments for the assessment of both prenatal and postnatal maternal distress. Another strength is that our sample was relatively similar to the population of pregnant women in the province of Quebec in terms of education level and median household income [42], supporting the generalization of the findings. However, the sample was not representative in terms of ethnicity (2.4% in the current sample vs 13% in the province of Quebec) [72]. One limitation is that we did not have data over the whole course of pregnancy or about breastfeeding, which would have allowed us to evaluate whether specific timing and duration of distress were associated with more negative outcomes, or whether the effect of postnatal distress was moderated by breastfeeding. The use of a parent‐report measure of infant development is also an important limitation since we cannot completely rule out the possibility that the strong association we observed between postnatal distress and infant development was partly accounted for by cognitive biases in distressed mothers. Also, it would have been interesting to include a sample of mother-infant dyads evaluated prior to the pandemic, to evaluate whether different associations would have been observed in both cohorts. Also, we did not have data on the degree of exposure to different stressors related to the pandemic in mother-infant dyads. Future research should look into the specific impacts of different COVID-19 stressors (financial, public health, loss of social support) on perinatal mental health and its link with infant development. Finally, the correlational design prevents us from establishing causal links.

Our findings have implications for clinical practices. During this pandemic, it is fundamental to implement clinical surveillance of mothers by questioning mood disorders and symptomatology in pregnant women and parents of a young child. Formal and informal support should also be offered to families, which has proven to be much needed during the transition to motherhood, and even more so during a pandemic [69]. In addition, online interventions should be offered to support maternal perinatal mental health.

Future research should use longitudinal designs to evaluate whether the impacts reported in our study persist in early childhood and later in life, and should use observational measures of infant development. The results also need to be replicated with high-risk samples and in other countries where public health measures are different.

## Supplementary Information

**Additional file 1:** **Table S1.** Pearson correlations between maternal perinatal distress and the subscales of the ASQ-SE:2.

**Additional file 2:** **Table S2.** A posteriori mean comparisons evaluating differences in infant socioemotional development according to the presence of clinically significant levels of prenatal and postnatal maternal distress.

## Data Availability

The datasets used and/or analyzed during the current study are available from the corresponding author on reasonable request.

## Abbreviations

COVID-19: Coronavirus disease 2019
M: Mean
Mage: Mean age
SD: Standard deviation
SE: Standard error
b: Non-standardized regression coefficient
β: Standardized regression coefficient
EPDS: Edinburgh Postnatal Depression Scale
K10: Kessler Psychological Distress Scale
ASQ-SE:2: Ages and Stages Questionnaire: Social-Emotional, Second Edition
SEM: Structural equation modeling
RMSEA: Root mean square error of approximation
CFI: Comparative fit index
SRMR: Standardized root square residual
CI: Confidence interval
ANOVA: Analysis of variance
11β-HSD2: 11β-Hydroxysteroid dehydrogenase
