# Supplementary material for: Socioemotional development in infants of pregnant women during the COVID-19 pandemic: the role of prenatal and postnatal maternal distress
Source: Child Adolesc Psychiatry Ment Health. 2022 Mar 31;16:28. doi: 10.1186/s13034-022-00458-x (PMC8969812; doi:10.1186/s13034-022-00458-x)
Supplement: Supplementary file 1 — Additional file 1: Table S1. Pearson correlations between maternal perinatal distress and the subscales of the ASQ-SE:2. [file 13034_2022_458_MOESM1_ESM.docx]

**Table S1.** Pearson correlations between maternal perinatal distress and the subscales of the ASQ-SE:2.

|  | 1 | 2 | 3 | 4 | 5 | 6 | 7 | 8 |
| --- | --- | --- | --- | --- | --- | --- | --- | --- |
| 1. EPDS total score (pregnancy) | - |  |  |  |  |  |  |  |
| 2. K10 total score (pregnancy) | .733** | - |  |  |  |  |  |  |
| 3. EPDS total score (2 months) | .434** | .457** | - |  |  |  |  |  |
| 4. K10 total score (2 months) | .466** | .555** | .736** | - |  |  |  |  |
| 5. Self-regulation | .017 | .037 | .158** | .157** | - |  |  |  |
| 6. Adaptive functioning | .078 | .080 | .154** | .152** | .337** | - |  |  |
| 7. Affect | .082 | .045 | .149** | .115* | .097* | .071 | - |  |
| 8. Social communication | .085 | .004 | .053 | .019 | .190** | .184** | .028 | - |
| 9. Interaction w/ parents | .019 | .048 | .166** | .133** | .288** | .225** | .088 | .395** |

Note. **p* < .05, ***p* < .001
