# Supplementary material for: Socioemotional development in infants of pregnant women during the COVID-19 pandemic: the role of prenatal and postnatal maternal distress
Source: Child Adolesc Psychiatry Ment Health. 2022 Mar 31;16:28. doi: 10.1186/s13034-022-00458-x (PMC8969812; doi:10.1186/s13034-022-00458-x)
Supplement: Supplementary file 2 — Additional file 2: Table S2. A posteriori mean comparisons evaluating differences in infant socioemotional development according to the presence of clinically significant levels of prenatal and postnatal maternal distress. [file 13034_2022_458_MOESM2_ESM.docx]

**Table S2.** A posteriori mean comparisons evaluating differences in infant socioemotional development according to the presence of clinically significant levels of prenatal and postnatal maternal distress

| Group comparison^a^ | | Mean difference | Standard error | p-value | Effect size^b^ |
| --- | --- | --- | --- | --- | --- |
| No distress^c^  (n = 267) | Distress at T1 only | -2.300 | 1.734 | 1.000 | -.154 |
|  | Distress at T2 only | -5.186 | 2.717 | .341 | -.348 |
|  | Distress at T1 and T2 | -7.089* | 2.112 | .005* | -.475 |
| Distress at T1 only (n = 112) | Distress at T2 only | -2.886 | 2.946 | 1.000 | -.193 |
|  | Distress at T1 and T2 | -4.789 | 2.388 | .273 | -.322 |
| Distress at T2 only (n = 29) | Distress at T1 and T2 | -1.903 | 3.185 | 1.000 | -.128 |

^a^ A posteriori comparisons of means using Bonferroni correction were performed following an ANCOVA controlling for infant age in weeks, *F*(3, 452) = 9.492, *p* < .001.

^b^ Effect sizes were calculated by dividing the difference between the adjusted group means by the pooled standard deviation.

^c^ Participants were considered as having significant distress when they had a score reaching the validated cut-off (≥ 30) of the Kessler Distress Scale and/or of the Edinburgh Depression Scale (≥ 11)

**p* < .001
